# Microplastic-driven carbon availability differentiates phage-host interactions in shaping soil resistome

**DOI:** 10.1093/ismejo/wrag258

**Published:** 2026-10-06

**Authors:** Lihong Xie, Lu Wang, Lijuan Ma, Jie Wang, Di Wu, Aijun Lin, Leyun Wang, Mao Ye, Xiujin Li, Tingting Zhang, Dong Zhu

**Affiliations:** Department of Environmental Science and Engineering, College of Chemical Engineering, Beijing University of Chemical Technology, Beijing 100029, China; State Key Laboratory of Regional and Urban Ecology, Institute of Urban Environment, Chinese Academy of Sciences, Xiamen 361021, China; Zhejiang Key Laboratory of Pollution Control for Port-Petrochemical Industry, CAS Haixi Industrial Technology Innovation Center in Beilun, Ningbo 315830, China; State Key Laboratory of Regional and Urban Ecology, Institute of Urban Environment, Chinese Academy of Sciences, Xiamen 361021, China; State Key Laboratory of Regional and Urban Ecology, Institute of Urban Environment, Chinese Academy of Sciences, Xiamen 361021, China; Institute for Global Food Security, School of Biological Sciences, Queen’s University Belfast, 19 Chlorine Gardens, Belfast BT9 5DL, United Kingdom; Department of Environmental Science and Engineering, College of Chemical Engineering, Beijing University of Chemical Technology, Beijing 100029, China; Department of Environmental Science and Engineering, College of Chemical Engineering, Beijing University of Chemical Technology, Beijing 100029, China; National Engineering Research Center for Soil Nutrient Management and Pollution Remediation, Institute of Soil Science, Chinese Academy of Sciences, Nanjing 210008, China; Department of Environmental Science and Engineering, College of Chemical Engineering, Beijing University of Chemical Technology, Beijing 100029, China; Department of Environmental Science and Engineering, College of Chemical Engineering, Beijing University of Chemical Technology, Beijing 100029, China; State Key Laboratory of Regional and Urban Ecology, Institute of Urban Environment, Chinese Academy of Sciences, Xiamen 361021, China; Zhejiang Key Laboratory of Pollution Control for Port-Petrochemical Industry, CAS Haixi Industrial Technology Innovation Center in Beilun, Ningbo 315830, China

**Keywords:** carbon availability, soil resistome, phage-host interactions, phage-augmented metabolism

## Abstract

The growing prevalence of microplastics (MPs) in agroecosystem has raised significant concerns regarding their ability in facilitating the dissemination of antibiotic resistance genes (ARGs). Biodegradable and nondegradable MPs exhibited inherently different degradation rates, resulting in divergent carbon bioavailability that might trigger distinct microbial response and ARG profiles. Here, by integrating multi-omics (metagenomics, virome, and metatranscriptomics) with experimental validation, we explored how microbial responses to MPs impacted ARG dissemination in a long-term field experiment. We showed that both biodegradable and nondegradable MPs significantly increased ARG abundance and transcriptional activity, differing in the drive mechanisms. Biodegradable MPs triggered bacterial oxidative stress and DNA damage response, increasing mobile genetic elements abundance and horizontal gene transfer of ARGs. Accordingly, increasing proportion of lysogenic phages and intensified phage-host interactions might promote ARG transduction through lysogenic conversion. In contrast, nondegradable MPs increased recalcitrant carbon, which enriched bacteria harboring genes for complex compound degradation. ARG-carrying bacteria within these taxa gained fitness advantages, facilitating their enrichment and ARG proliferation. Concurrently, phages infecting ARG-carrying bacteria encoded more auxiliary metabolic genes in complex carbohydrate metabolism, thereby enhancing host competitiveness and promoting ARG spread. In vitro validation experiments confirmed that lysogenic phages facilitated transduction of ARGs under biodegradable MPs, while phage-encoded auxiliary metabolic genes conferred growth advantages on ARG-carrying bacteria utilizing recalcitrant carbon source, consistent with conditions induced by nondegradable MPs. Our findings clarify how phages differentially impacted ARG profiles under biodegradable and nondegradable MPs, underscoring the importance of considering phage-mediated processes in assessing the risks of MPs and ARG dissemination.

## Introduction

Antibiotic resistance constitutes a critical threat to global health and food security [1, 2]. Antibiotic resistance genes (ARGs) encoding functionally diverse resistance determinants are now ubiquitous across natural and engineered ecosystems [3]. Their proliferation compromises the clinical efficacy of essential antibiotics, posing a threat to global infectious disease management [4]. Soils have emerged as a principal reservoir of ARGs, particularly where intensive plastic product inputs (e.g. plastic film mulching) impose chronic selective pressures that favor resistance proliferation [5, 6]. Microplastics (MPs) exposure could exacerbate horizontal transfer of ARGs by increasing conjugative transfer frequencies [7]. A nuanced perspective highlight the core role of microbial interactions and spatial structure induced by soil carbon availability in ARG profile [8]. As carbon-based polymers, MPs also alter soil carbon pools [9]. Owing to differences in their degradability, biodegradable, and nondegradable MPs differentially affect soil dissolved organic matter, thereby exerting distinct regulatory effects on soil carbon availability [10]. However, the specific links between MP-driven carbon alteration and ARG proliferation remain unclear.

Nondegradable plastics such as polyethylene and polypropylene exhibit strong chemical stability, resisting microbial degradation and contributing little bioavailable carbon to surrounding soil [11]. Nondegradable MPs function as persistent ecological refugia, where stable surfaces attachment facilitates biofilms formation, and the associated carbon limitation co-select for specific ARG-carrying taxa [12, 13]. Biodegradable MPs undergo accelerated disintegration, generating smaller fragments, and creating continuously degrading surfaces that promote more rapid and unstable microbial succession [14]. This degradation process releases bioavailable carbon that stimulates bacterial growth and metabolic activity, potentially enhancing bacterial interactions and contact frequency, thereby accelerating horizontal gene transfer [15]. MPs increased abundance of ARGs through broadening their bacterial host range [16], elevating the probability of horizontal gene transfer [17] and intensifying co-selection pressure [18]. And the relative contribution of these processes might vary depending on the different types of MPs. Biodegradable MPs may shape ARG profiles by releasing bioavailable carbon that fuels carbon-driven microbial responses [15], whereas nondegradable MPs impose carbon limitation and associated selective pressure [19]. However, how the MP-induced carbon availability differences influence the bacterial community and functional restructuring, and consequently lead to divergent ARG profiles, needs in-depth exploration.

Bacteriophages (hereinafter referred to as phages) are increasingly recognized as important vectors for ARGs [20]. Phages serve as mobile genetic elements (MGEs) that facilitate cross-host transfer of ARGs through transduction, and have been found to harbored diverse ARGs in human-impacted environments [21, 22]. Recent research revealed that phages influenced MP-driven ARG dissemination through horizontal gene transfer and phage lysis, underscoring their contribution to ARG proliferation [23]. Beyond direct gene transfer, phages exert profound control over the structure, function, and eco-evolutionary dynamics of soil microbiomes through phage-host interactions [24, 25]. For example, lysogenic phages enlarges the functional genes pool through lysogenic conversion, including ARGs, to enhance host adaptive potential [26]. Furthermore, phages also encode auxiliary metabolic genes (AMGs) that reprogram host metabolism, thereby enhancing adaptive resilience and indirectly increasing the abundance of ARG-carrying hosts and ARGs under resource stress [27, 28]. MPs introduce novel solid-phase interfaces and differential bioavailable carbon inputs, which can alter the ecological niches of both bacteria and their associated phages [29], and are expected to reshape these intricate phage-host interactions [30]. Previous studies mainly focused on the bacterial responses to MPs and their contribution to ARG enrichment, while the phage-mediated dissemination remaining largely unexplored. MP-induced perturbations in carbon availability may selectively enrich for phages harboring ARGs or AMGs, potentially resulting in divergent pathways of ARG alterations under biodegradable and nondegradable MPs conditions.

To explore whether how different types of MPs affect the dissemination of ARGs in soil via phage-host interactions, we conducted a 2-year field experiment treated with four commonly used biodegradable MPs [polylacticacid (PLA), polyhydroxyalkanoates (PHA), Polybutylene succinate (PBS), and Polybutylene adipate-coterephthalate (PBAT)] and nondegradable MPs [polyvinylchloride (PVC), polystyrene (PS), polypropylene (PP), and polyformaldehyde (POM)], respectively. Metagenomics, viromics, and metatranscriptomics analysis were employed to characterize the response of phage-host interactions, microbial community dynamics, and gene expression to MPs pollution. Our work aims to reveal how biodegradable and nondegradable MPs differentially induce bacterial and phage responses, and to link these distinct patterns to variations in soil ARGs. We hypothesize that the contrasting degradability between biodegradable and nondegradable MPs drives distinct patterns of ARG variations: biodegradable MPs facilitate ARG proliferation via increased lysogenic phages and enhanced lysogenic conversion, while nondegradable MPs enrich competitive ARG-carrying taxa and boost their fitness through phage-encoded AMGs under resource-limited condition. To verify this, we conducted two validation experiments, isolation of lysogenic phages and functional gene synthesis, to valid the different main contributing factors on ARGs variations. The proposed conceptual framework will offer pivotal insight for assessing and mitigating the risks of MP-driven ARG dissemination under increasing plastic pollution.

## Results

### The influence of MPs on ARGs encoded by bacteria and phage in soil

A profile of ARGs across all soil samples comprised 23 types and 484 subtypes, of which the most diverse ARG type was identified as multidrug resistance genes containing 76 subtypes, followed by macrolide-lincosamide-streptogramin (MLS), vancomycin, and bacitracin resistance genes (Fig. S1). Notably, the total abundance of ARGs encoded by bacteria was significantly higher in MPs-treated soils (*P* < .05), especially in soils treated with PBAT, POM, PP, and PS (Fig.  1A). The ARGs mainly conferred resistance to multidrug, MLS, and vancomycin, with an average ratio of 24.1%, 20.7%, and 15.0%, respectively. While the abundance of ARGs encoded by phages were significantly enriched in soils treated with PBAT, POM, PS, and PVC (*P* < .05) (Fig.  1B). These ARGs were dominated by multidrug, MLS, and aminoglycoside, with an average of 27.3%, 25.9%, and 16.8%, respectively. Moreover, compared to the control (CK, soil without MP addition), the number and types of active ARGs encoded by bacteria and phages increased in soils with MPs (Fig. S2, bubble plot), along with the enhanced transcriptional activity of ARGs in the soil with MPs (Fig. S2, heatmap, Supplementary Tables 1 and 2). Specifically, the addition of biodegradable MPs mainly increased the activity of ARGs encoding resistance to tetracycline; while nondegradable MPs treatment enhanced the activity of ARGs responsible for resistance to multidrug and MLS. Importantly, for the ARGs encoded by bacteria, nondegradable MPs had a significant effect on the total abundance of active ARGs (*P* < .01, Fig.  1C). For the ARGs encoded by phage, significant enrichment in the total abundance of active ARGs occurred in the soil treated with MPs, especially in biodegradable MPs (*P* < .01, Fig.  1D). Additionally, 47 and 13 high risk ARGs classified as Rank-I and Rank-II were identified across all soil samples, respectively (Supplementary Table 3). The introduction of different types of MPs resulted in varying ecological risk. Specifically, the biodegradable MP group showed significant enrichment of ARGs classified as Rank-I risk (*P* < .05, Fig. S3A), while the nondegradable MP group showed preferential accumulation of ARGs classified as Rank-II risk (*P* < .05, Fig. S3B).

**Figure 1:**
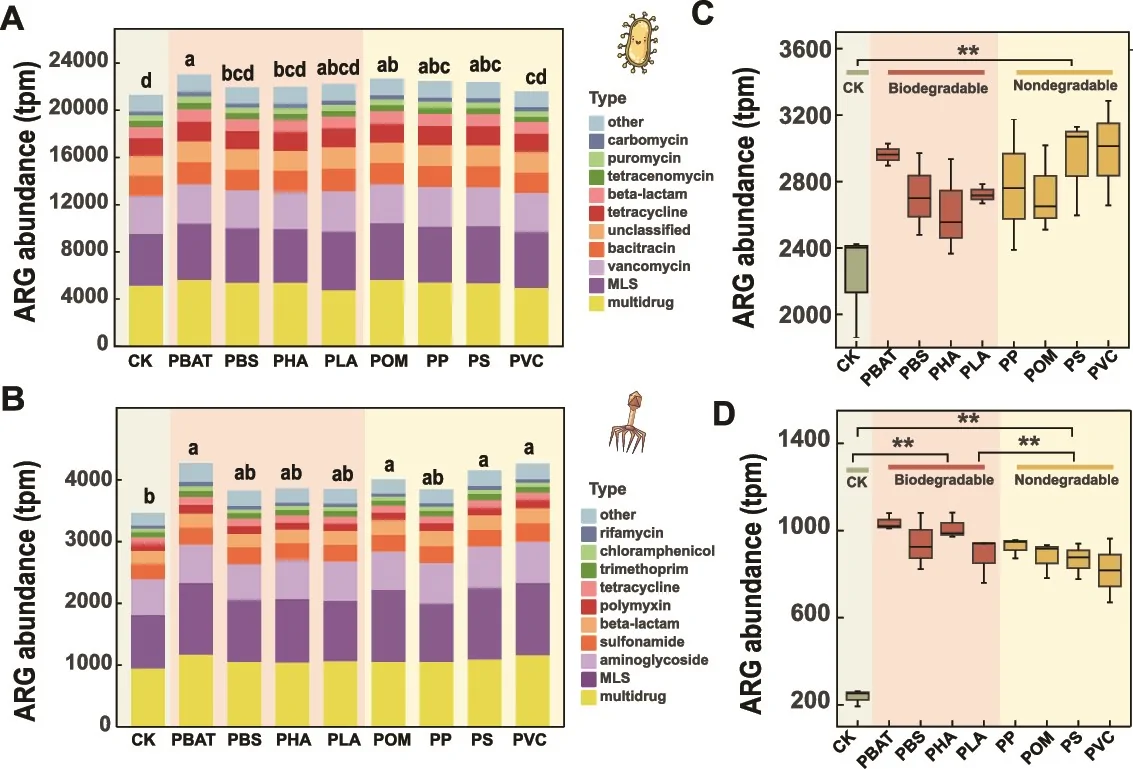
The variations of ARGs in soil under the influence of MPs. Abundance of ARGs classes in soil bacteria (A) and phage (B). The abundance of active ARGs in bacteria (C) and phage (D). Different lowercase letters above the box and stack bars indicate significant differences among different treatment levels in Tukey’s post hoc tests (*P* < .05). For boxplots, asterisks indicate significant differences between any two treatments (*t* test, ^*^P < .05, ^**^P < .01).

We identified 51 subtypes and 7 types MGE that could facilitate the prevalence and persistence of soil ARGs through horizontal gene transfer. And linear regression analysis showed a significant positive correlation between MGE and ARGs (R^2^ = 0.292, *P* < .05, Fig. S4A). The highest total abundance of MGE was observed in soils treated with biodegradable MPs (*P* < .05) (Fig. S4B).

### Main drivers of ARGs patterns under different MPs

The introduction of biodegradable MPs, including PLA and PBAT, significantly increased DOC content, with pronounced effects on SOC in PHA and PBS treatments. In contrast, nondegradable MPs reduced SOC levels in soils with PP and decreased DOC concentrations in soils with PVC and PS (Fig. S5). Meanwhile, the introduction of MPs led to the imbalance nutrition, the C/N increased significantly in the biodegradable MPs treatments (*P* < .05) (Fig. S5). The bacterial composition and community varied in response to MPs exposure. At phylum level, the relative abundance of Actinobacteriota decreased from 19.64% in CK to 15.33%, 16.16%, 16.73%, and 18.96% in POM, PP, PS, and PVC treatments, respectively (Fig. S6), while the relative abundance of Acidobacteriota increased from 13.78% in CK to 14.22%, 14.29%, 13.86%, and 14.64% in PBAT, PBS, PHA, and PLA, respectively. At family level, there was a significant increase in the relative abundance of Xanthobacteraceae (Fig. S7).

Random forest was conducted to assess the contribution of soil properties, bacterial community, and MGE abundance on ARG abundance under biodegradable and nondegradable MPs exposure, respectively (Fig.  2A and B). Under biodegradable MPs exposure, MGE abundance explained the greatest proportion of the variance in ARG enrichment (54.4% increase in MSE upon permutation) (Fig.  2A), whereas bacterial community dissimilarity emerged as the dominant driver in the presence of nondegradable MPs (45.7% increase in MSE) (Fig.  2B).

**Figure 2:**
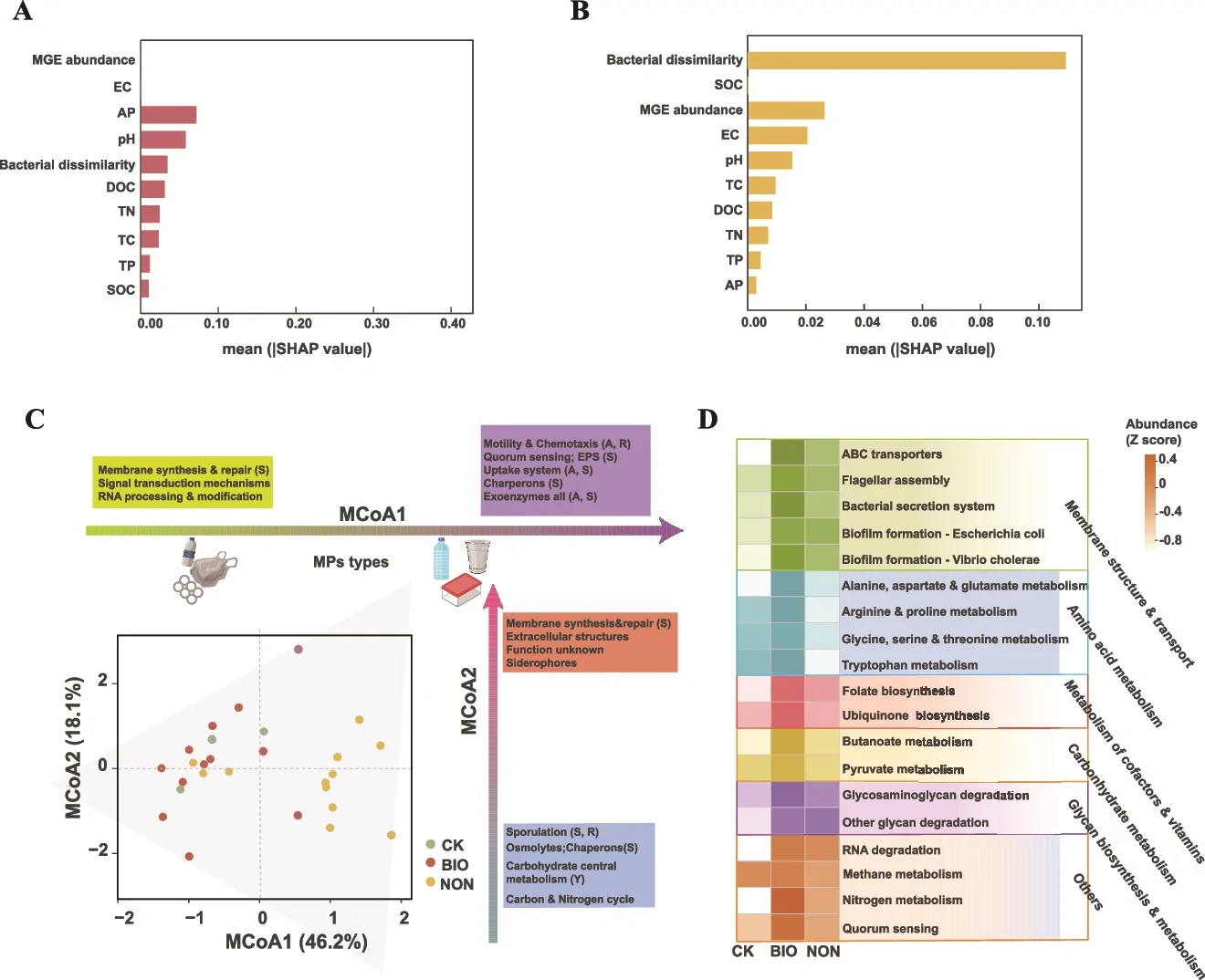
Analysis of distinct driving factors of ARG enrichment. Random forest analysis reveals the main factors in biodegradable (A) and nondegradable (B) MPs treatments. The life history strategy of bacteria under biodegradable and nondegradable MPs treatments (C). The relative abundance of bacterial features response for antibiotic resistance pathways derived from KEGG C level (D).

The different types of MPs also drove variations in bacterial life history strategy. As profiled by MCOA analysis, the first dimension (MCOA1) and second dimension (MCOA2) explained for 46.2% and 18.1% of the variances, respectively (Fig.  2C). In soil with biodegradable MPs, bacterial communities progressively shifted toward the upper end of MCOA1. Genes related to chemotaxis, motility, and uptake system were highly expressed for optimizing resource acquisition under biodegradable MPs exposure. Meanwhile, bacteria exhibited a higher relative abundance of genes associated with extracellular polymeric substances (EPS) production and quorum sensing, key components of stress tolerance strategies (Fig. S8). The transcriptional activity of complex carbon degradation genes was positively correlated with carbon availability (R^2^ = 0.2171, *P* < .05, Fig. S9). The heatmap showed the KEGG pathways associated with biofilm formation, membrane transport, and metabolism of cofactors were upgraded in soil with MPs, especially with the addition of biodegradable MPs (Fig.  2D). These pathways are key factors in the proliferation of antibiotic resistance. Furthermore, the gene related to SOS response, including *oxyN* (Biodegrdable vs. CK: *P* < .05), *uvrA* (Biodegrdable vs. CK: *P* < .05), *uvrB, uvrC,* and *recA*, were highly expressed in soil with MPs exposure. Meanwhile, the addition of MPs led to enrichment of genes involved in ROS generation in soil with biodegradable MPs, including catalases *katG* (Biodegradable vs. CK: *P* < .05), hydroperoxide reductase *ahpA* and *aphB* (Fig. S10A). Additional ROS-related genes, such as *SOD2, SOD1, rpoS,* and *rpoH*, conjugative transfer gene, such as *traR*, upgraded in soil with MPs. Accordingly, the transcriptional activity of genes related to oxidative stress and SOS response was significantly higher compare to CK (Biodegradable vs. CK: *P* < .05, Fig. S10B, Supplementary Table 4).

### Effects of MPs on soil phage

A total of 76 high-quality metagenome-assembled genomes (MAGs) were recovered from all soil samples (Supplementary Table 5), among which 23 MAGs were identified as antibiotic-resistant bacteria based on the presence of ARGs (Fig.  3A). A total of 1960 viral operational taxonomic units (vOTUs) were identified in all soil samples, among which Caudovirales were the most abundant viral order (Supplementary Table 6, Fig. S11A). The relative abundance of Caudovirales increased from 32.12% in CK to 44.91%, 43.18%, 37.64%, 45.98%, 31.75%, 30.15%, 36.50%, and 46.89% in PBAT, PBS, PHA, PLA, POM, PP, PS, and PVC, respectively. The phage diversity was significantly increased under biodegradable MPs treatments (*P* < .05), which was positively correlated with carbon availability (R^2^ = 0.21, *P* > .05, Fig. S11). And 61 of the total MAGs were identified as the hosts of phage, of which Actinobacteriota (65.22%), Acidobacteriota (13.04%), and Proteobacteria (8.7%) were the main viral host. The relative abundance of phages with predicted host bacteria increased in MPs treatments. Specifically, the relative abundance of phages hosted by Actinobacteriota increased from 5.73% to 10.24%, 7.93%, 9.2%, and 7.53% in PBAT, PBS, PHA, and PLA treatment, respectively. While phages hosted by Acidobacteriota increased from 2.2% to 4.01%, 5.37%, 4.47%, and 5.22% in POM, PP, PS, and PVC treatment, respectively (Fig.  3A). Among all identified viral contigs, 86 vOTUs were found to harbor 209 distinct ARGs. Host prediction analysis revealed that these ARG-carrying viral contigs primarily infected hosts from three bacterial phyla: Actinobacteriota (43.2%), Proteobacteria (32.1%), and Acidobacteriota (13.6%) (Fig.  3B).

**Figure 3:**
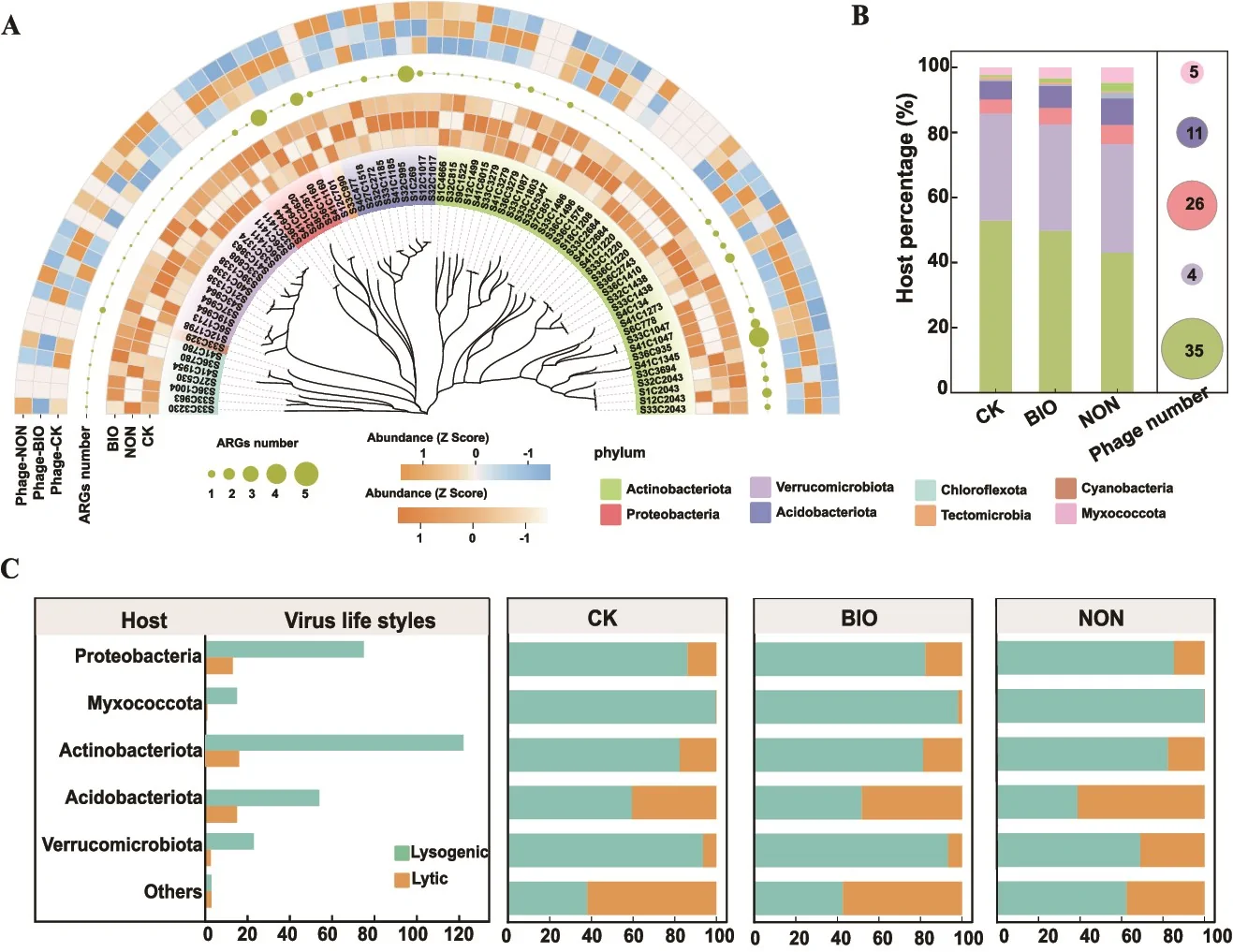
The profile of soil phage. (A) The information of MAGs, including the relative abundance, the number of ARGs per MAG, and the abundance of phage hosted by each MAGs. (B) The stack bar chart shows the proportion of hosts that harbor phage encoding ARGs. (C) The ratios of lytic phages hosted by specific bacterial phyla, the variations of phage life style across MPs treatments.

The viral lifestyle was altered owing to the introduction of MPs. The proportion of lytic phages markedly increased from 19.40% to 22.23% and 31.27% in biodegradable and nondegradable MPs treatments, respectively. Specifically, the proportion of lytic phages hosted by Acidobacteriota increased from 40.45% to 48.13% and 61.09% in biodegradable and nondegradable MPs treatments, respectively (Fig.  3C).

### Effects of different soil phage-host interactions on ARGs variations

Among the ARGs encoded by phage, lifestyle annotation indicated that over 88% of ARG-harboring vOTUs contained lysogenic markers, significantly outnumbering those with lytic signatures (Fig. S12). Notably, the total abundance of ARGs encoded by lysogenic phages was significantly increased in biodegradable MPs treatments (biodegradable vs, CK: *P* < .05, Fig.  4A). Meanwhile, the phage-host interactions were significantly altered due to the addition of biodegradable MPs, which was proved by the significant increment of relative abundance of phages that harbored ARGs (biodegradable vs. CK: *P* < .01, Fig.  4B). Concurrently, significant linear correlation between the relative abundance of phages and bacterial hosts was also observed in biodegradable MPs treatments (*R*^2^ = 0.35, *P* = .04, Fig.  4B). While phage-host coupling was not significant in nondegradable MPs treatments (*R*^2^ = 0.23, *P* = .1400, Fig. S13). The abundance of phages that harbored ARGs varied under MPs exposure. Specifically, phages with increased abundance encoded a higher proportion of ARGs in biodegradable MPs treatments. Among the phages encoding ARGs, the viral abundance increased by 60.5% and 48.8% under exposure to biodegradable and nondegradable MPs, respectively, with encoding 54.15% and 50.1% of the ARGs (Fig.  4D).

**Figure 4:**
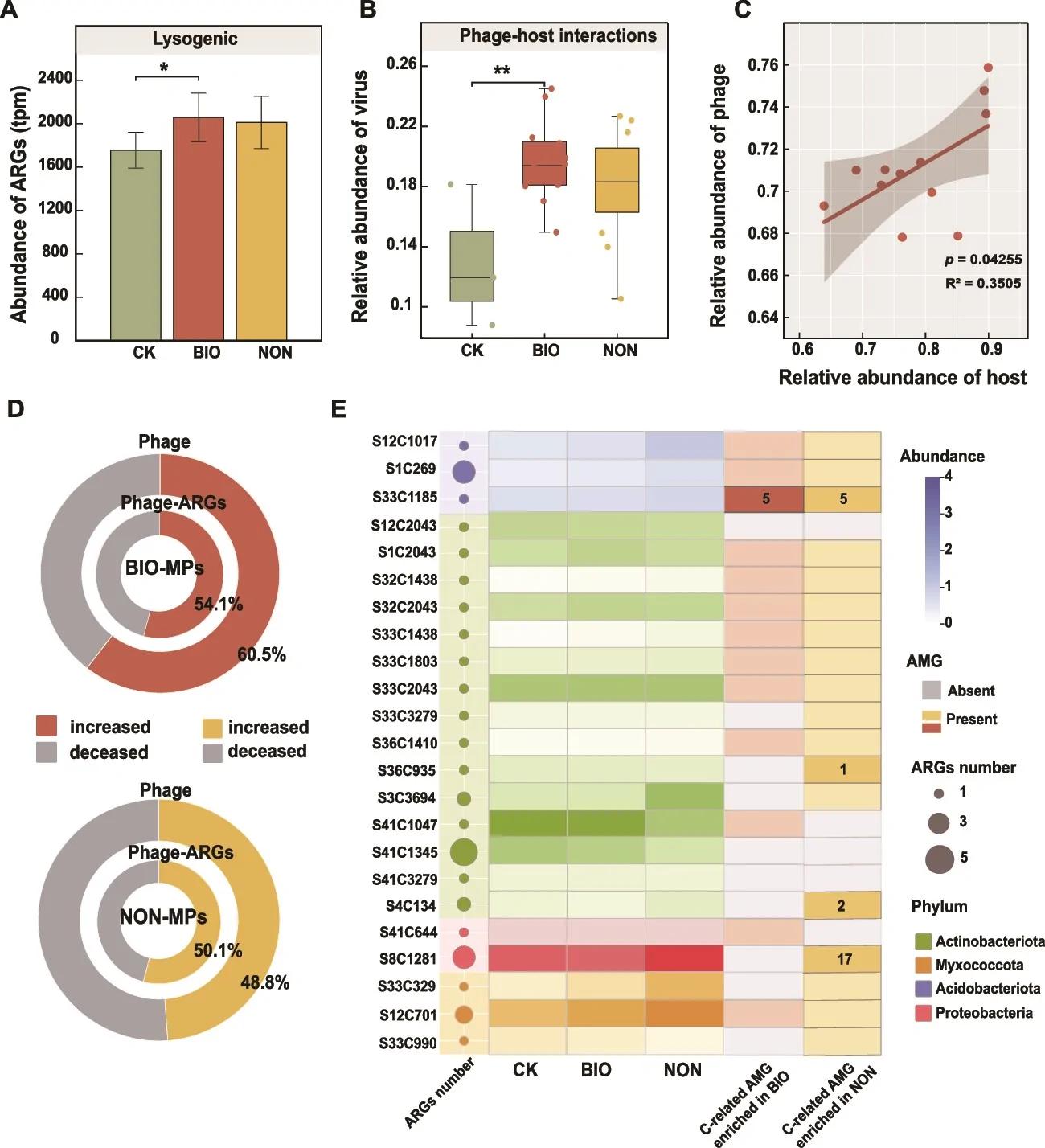
The variations of phage-encoded ARGs and AMGs. (A) The variations of abundance of ARGs encoded by lysogenic phage. The variations of phage-host interactions across MPs treatments, characterizing by the relative abundance of phage with predicted hosts (B), as well as the linear correlation between phage and its host under biodegradable MPs (C). Abundance variations of phage (compared to CK, outer circle), and the abundance variation of ARGs that harbored in phage with increased abundance (inner circle) under biodegradable and nondegradable MPs treatments (D). Heatmap of ARG-carrying bacteria abundance (left) and number of carbon-related AMGs in phages infecting enriched ARG-carrying bacteria (right) (E). *P*-values were calculated by *t* test. Significance among treatments is denoted as ^*^P < .05, ^**^P < .01.

Exposure to MPs led to an increase in the abundance of ARG-carrying bacteria with 13 and 17 being identified under biodegradable and nondegradable MPs treatments, respectively (Fig. S14, outer circle). Among the ARGs-carrying bacteria that showed increased abundance, Actinobacteriota was the dominant phylum, comprising 61.5% and 64.7% of the total in biodegradable and nondegradable MPs treatments, respectively (Fig. S14, inner circle). Besides, the number of complex carbon degradation genes annotated in KEGG and CAZy database was significantly higher in ARGs-carrying bacteria (*P* < .01, Fig. S15A and B), such as glycosyl hydrolase family 6, glycosyl hydrolase family 7 involved in cellulose degradation, glycosyl hydrolase family 76, glycosyl hydrolase family 26 and glycosyl hydrolase family 133 involved in mannan degradation, *SGA1* and *CELB* involved in polysaccharides metabolism. Moreover, the transcriptional activity of these genes was significantly higher in ARGs-carrying bacteria (*P* < .001, Fig. S15C and D, Supplementary Table 7).

We further analyzed phage-encoded AMGs related to complex carbohydrates degradation through VIBRANT and DRAMV. A number of functional genes, including cellulase, glycosyl hydrolase family 16 and glycosyl hydrolase family 71 involved in β- glucan degradation, glycosyl hydrolase family 43 involved in β-xylan degradation, were annotated by Pfam and CAZy database. The addition of different MPs types altered the composition and function of AMGs in soil. Under nondegradable MPs treatment, phages harbored by enriched ARGs-carrying bacteria encoded more AMGs related to complex carbohydrates metabolism (biodegradable vs nondegradable = 5: 25), helping host survive in carbon limited conditions (Fig. 4E). Among them, proteobacteriophage (S8C1281) showed the highest abundance under nondegradable MPs exposure, encoding 17 AMGs in associated phages.

### Experimental verification of the role of phage in ARGs dissemination under MPs exposure

To validate the role of lysogenic phages on the ARGs proliferation under biodegradable MPs exposure, we extracted the bacterial and phage community from soil with and without biodegradable MPs treatments (Fig.  5A). The results confirmed the enhanced transduction ARGs mediated by lysogenic phage. The relative abundance of ARGs were significantly higher in bacteria-lysogenic phage (BP) treatment than bacteria (B) treatment or bacteria-inactivated phage (BIP) treatment in both two soils (*P* < .05). Additionally, the overall abundance of ARGs were higher in soil with biodegradable MPs addition than CK, especially in the bacteria-lysogenic phage treatment (Fig.  5C).

**Figure 5:**
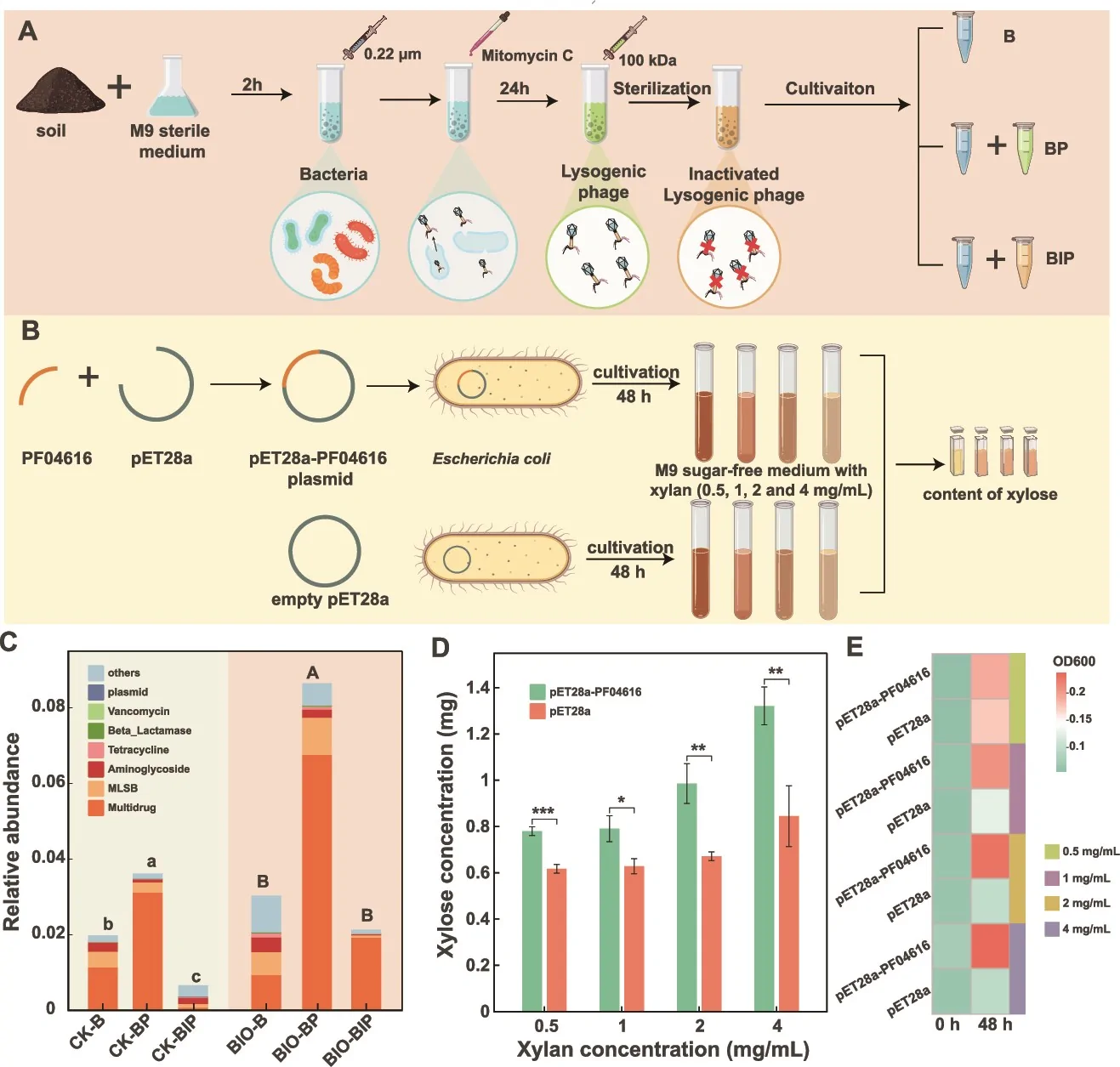
Experimental validation of distinct phage-host processes under biodegradable and nondegradable MPs. (A) Workflow for an in vitro cultivation experiment to valid the role of lysogenic phage in ARGs dissemination in carbon-rich condition. (B) Workflow of gene synthesis and poly saccharide degradation experiment to valid whether phage assist host carbon utilization in carbon-limited condition. (C) Effects of lysogenic phages on the relative abundance of ARGs between biodegradable MPs treatments and CK. (D) The concentration of xylose degraded by *E. coli* carrying pET28a-PF04616 and pET28a. (E) Heatmap showed the OD_600_ of in the cultivation experiment.

To validate the ability of phage-encoded AMG to assist the host in utilizing complex carbon sources under source-limited conditions, we employed xylan as the sole carbon source to explore the degradation efficiency by *Escherichia coli* (*E. coli*) carrying pET28a-PF04616 (Fig.  5B). In media with different concentrations of xylan, *E. coli* carrying pET28a-PF04616 exhibited significantly higher degradation ability of xylan, proved by a higher concentration of degradation product (ie, xylose) (Fig.  5D  *P* < 0.05). Meanwhile, the results of OD_600_ also showed that the *E. coli* carrying pET28a-PF04616 grew better after 48 h, indicating that PF04616 assisted host bacteria in surviving under source-limited conditions (Fig.  5E).

## Discussion

### ARGs proliferation and ecological risks under MPs exposure

Our research showed a significant enrichment of total and active ARGs in both bacteria and phage under typical biodegradable and nondegradable MPs treatments compared to control. This indicated MPs could enhance dissemination potential of ARGs in soil. The enriched ARGs, such as *mexB* and *mexF*, were involved in bacterial efflux pump systems, potentially confer increased antibiotic resistance through expelling active and rapid compounds expel [31]. Concerningly, the addition of MPs increased the risk of ARGs, including these ARGs, notably enriching high risk ARGs commonly associated with human pathogens, raising concerns about potential human health impacts through food-chain transfer [32]. Recent research showed that biodegradable MPs exhibited greater ARG enrichment than conventional MPs in soils [6], aquatic [33] and wetland ecosystems [34]. In our research, biodegradable MPs significantly enriched ARGs classified as Rank I (e.g. *mdtL, rmtG*), while nondegradable MPs significantly enriched ARGs classified as Rank II. A preferential increase in transcriptional activity was also observed for tetracycline ARGs (biodegradable MPs) and beta-lactam ARGs (nondegradable MPs). This pattern may be associated with the enrichment of Actinobacteriota and Acidobacteriota—the primary host phylum of these resistance genes, respectively. These findings suggested that assessing the environmental safety of biodegradable MPs may require consideration of not only their biodegradability but also their potential impacts on the microbial ecosystem during the degradation.

### Distinct bacterial process driving ARGs enrichment under MPs

However, the main drivers affecting ARGs enrichment may be distinct between non- and bio-degradable MPs. Our results revealed that the nondegradable MPs increased ARGs in soil through selectively enrichment of ARGs-carrying bacteria: The bacterial community dissimilarity was the most important factor contributing to ARGs enrichment under long-term exposure of nondegradable MPs (Fig.  2B). These community variations can be attributed to the selective enrichment of ARGs-carrying bacteria. Functional gene analysis revealed the enrichment of genes involved in resources acquisition (i.e. chemotaxis, motility), substrate retention (i.e. EPS secreting) [35], and complex compounds degradation [30]. This suggests that, in response to the decrease in soil bacterial carbon availability induced by the addition of nondegradable MPs (Fig. S5), the bacteria community might adapt by enhancing its capacity to acquire related gene (Fig.  2C) [30]. In this context, in addition to exploitative competition through the selection of specific metabolic genes, bacteria might also gain a competitive advantage by developing antibiotic resistance, i.e. interference competition, which might explain the enrichment of ARGs-carrying bacteria [36]. This was evidenced by the observation of a greater number of ARGs-carrying bacteria with increasing abundance in nondegradable MPs treatments (Fig. S14). This interference competition might be intensified under carbon-poor conditions, where the production of antibiotics would eliminate surrounding competitors, and the killed bacteria was also a source of carbon for the resistant population [37].

In this research, we observed a higher abundance and transcriptional activity of genes related to cellulose degradation, mannan degradation, and polysaccharide metabolism within ARGs-carrying bacteria (Fig. S15). This suggested that these ARGs-carrying bacteria, which possessed the ability to utilize complex compounds, could provide extra competitive fitness, thereby promoted the dissemination of ARGs [37]. Consistently, the enrichment of specific ARG-carrying taxa, such as Xanthobacteraceae family, corroborated the findings from amplicon sequencing. These taxa have been previously reported to harbor diverse ARGs, conferring aminoglycoside, and multidrug resistance [38]. Collectively, under nondegradable MPs exposure, available carbon limitation and intensified competition might drive the selective enrichment of ARG-carrying bacteria. These taxa harbored genes for complex compound degradation, potentially facilitating their proliferation, and increasing overall ARGs abundance (Fig.  6).

**Figure 6:**
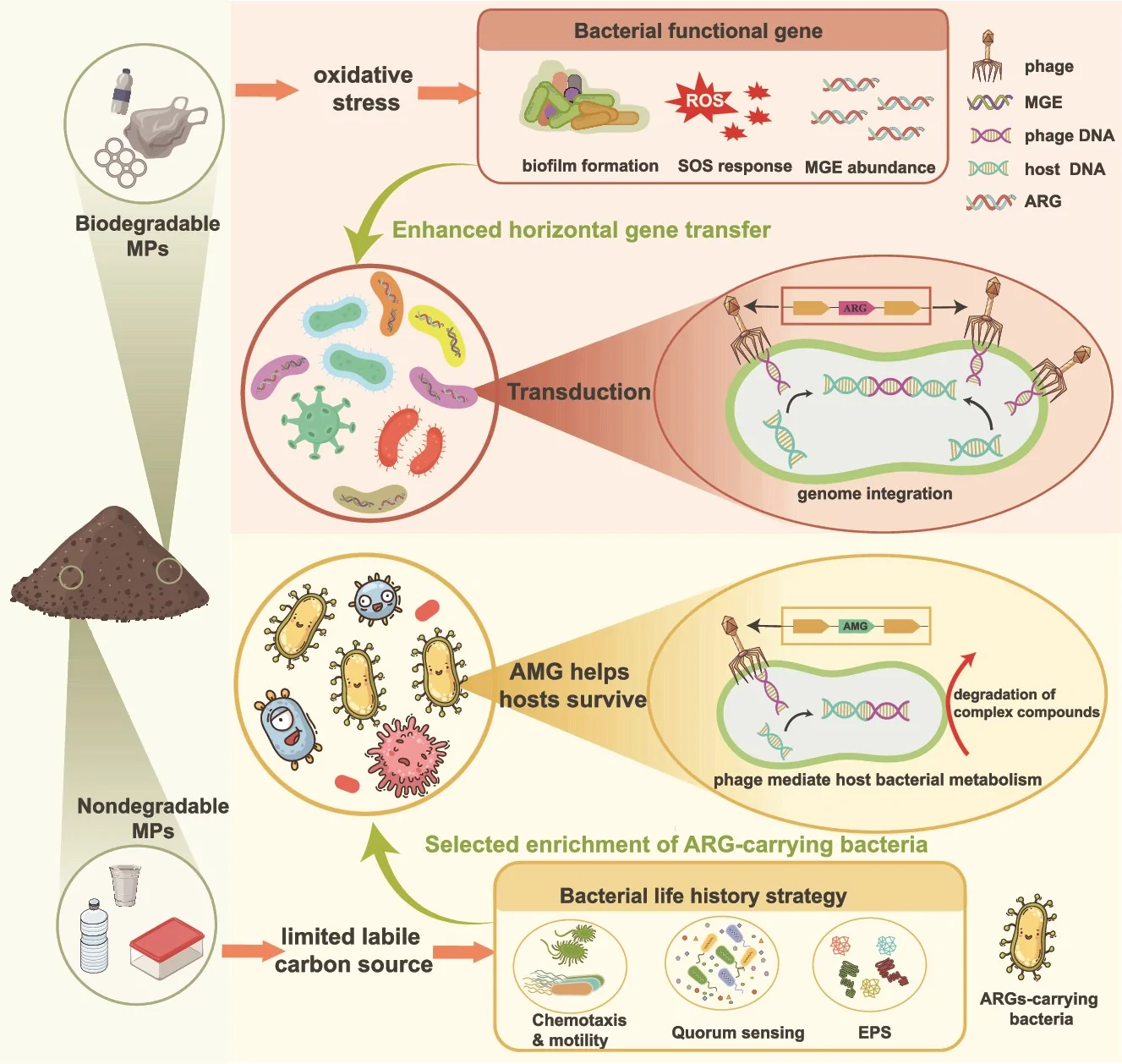
Divergent facilitation mechanisms of ARGs dissemination under biodegradable and nondegradable MPs exposure: Comparative roles of bacterial and viral communities.

Under exposure to biodegradable MPs, the increase in ARGs may be caused by increased horizontal gene transfer at the community level, rather than enrichment of specific bacterial taxa. This result is proved by the significantly increased abundance of MGEs (Fig. S4B), along with the positive correlation between MGEs and ARGs (Fig. S4A). Random forest analysis also indicated that the abundance of MGEs was the main driving factor for the enrichment of ARGs under biodegradable MPs treatments (Fig.  2A). As a fundamental engine of microbial evolution, horizontal gene transfer operates in concert with mutational processes and selective pressures to enhance adaptive responses to fluctuating environmental conditions [39]. This change can also be demonstrated by the increased abundance and transcriptional activity of genes related to SOS response (Fig.  2D, Fig. S10). The increase in MGEs abundance appeared to be regulated by bacterial oxidative stress [40], and serves as a key driver for bacterial plasmid conjugation, significantly promoting the horizontal transfer of ARGs even in the absence of antibiotic selection pressure [41]. The activation of the SOS response removed inhibition of the core gene (e.g. *LexA*) on some MGEs, thus facilitating the horizontal gene transfer of ARGs through the elevated MGEs abundance [42, 43].

During the degradation of biodegradable MPs, the release of oxidation byproducts such as plastic additives (e.g. phthalates) induces bacterial stress responses [44]. Meanwhile, the degradation of biodegradable MPs releases organic compounds at a faster rate than that of conventional MPs [45]. Prolonged exposure to biodegradable MPs may gradually exacerbate the C/N imbalance (Fig. S5C) and potentially also trigger bacterial stress responses [46]. Moreover, the variations in bacterial functional genes would also indirectly promote the dissemination of ARGs. Specifically, the significant enrichment of pathways, membrane structure, and function (e.g. ABC transporters, flagellar assembly), biofilm formation, metabolism of cofactors (Fig.  2D), provided environmental conditions for MGE-mediated ARGs transfer [47, 48]. Besides, the upregulation of the conjugation transfer genes could act synergistically to accelerate ARGs spread through increased membrane permeability and enhanced bacterial conjugation efficiency [49, 50]. Collectively, biodegradable MPs might promote the increase of MGE abundance by inducing bacterial stress responses, thereby enhancing the MGE-mediated horizontal transfer process and driving the enrichment of ARGs (Fig.  6).

### Distinct contribution of phages in ARGs enrichment

We found that under MPs pollution, viruses not only increase the abundance of ARGs encoded by themselves, but also promote the spread of host ARGs through lifestyle alteration and AMGs. Both biodegradable and nondegradable MPs increased the abundance and activity of phage-encoded ARGs (Fig.  1B and D). Biodegradable MPs triggered higher abundance of ARGs harbored by lysogenic phages (Fig.  4A), suggesting that lysogenic phages might actively contributed to ARGs transduction and promoting host adaptation and resistance [51]. Lysogenic phages facilitate ARG transfer more efficiently than lytic phages via prolonged host genome integration (i.e. lysogenic conversion) [28]. Consistently, phages, as vectors of MGEs, could promote ARGs dissemination through transduction [52]. These observations were consistent with the finding that horizontal gene transfer mainly facilitated the ARGs enrichment in the biodegradable MPs treatments. In addition, the strengthened phages-host interactions in biodegradable MPs treatments may be attributed to its higher lysogenic ratio (Fig.  3C). Therefore, the increased abundance of lysogenic phages and strengthened phages-host interactions might jointly be associated with the ARGs proliferation under biodegradable MPs. Lysogenic phages integrated their genomes into the host genome, facilitating the cooperative maintenance of community stability and highlighting the crucial role of phages in bacterial adaptation to environmental stress [22]. Moreover, the observed intensification of host-phage gene exchanges also explained the increased proportion of ARGs carried by phage genomes under biodegradable MPs treatment (Fig.  4D). Our in vitro incubation experiments further supported this hypothesis: lysogenic phages isolated from soils with biodegradable MPs significantly increased the absolute abundance of ARGs, compared with phages from control soils (Fig.  5C).

On the contrary, the proportion of lytic phages was significantly higher under nondegradable MP exposure (Fig.  3C, increased from 19.40% to 31.27%) reflecting the adaptive capacity of phages under reduced available carbon sources [30]. Phage-mediated bacterial lysis and subsequent cell death might also account for the distinct bacterial community structures observed in the nondegradable MPs treatment. Meanwhile, lysogenic phages that remained dominated in nondegradable MPs treatments encoded AMGs related to complex carbohydrate metabolism (Fig.  4E), exhibiting another adaptive strategy to resource limitation. Phages transferred AMGs to the host, assisting the host in the utilization of limited resources; in turn, the dominant population status of host bacteria benefited the continuous proliferation of phages [28, 29], thereby facilitating ARGs dissemination. Consistent with this, a higher proportion of ARGs-carrying bacteria exhibited increased abundance under nondegradable MPs exposure (Fig.  4D and E), supporting the role of phage-encoded AMGs in driving the proliferation of ARGs-carrying bacteria and the transmission of ARGs under MPs stress. This effect was most pronounced in Proteobacteria and their associated phages (proteobacteriophages), which carried a greater number of these AMGs. Notably, the S8C1281, an ARGs-carrying bacteria of Proteobacteria, carried the highest number of AMGs and exhibited the most substantial population growth (Fig.  4E), further supporting the role of phage-encoded metabolic genes in driving ARGs-carrying bacteria proliferation and, consequently, ARG dissemination under MPs stress. For example, glycosyl hydrolases family 43 was striking at bio-functionality that exhibited both β-xylosidase and α-arabinofuranosidase activities [53]. Phages can enhance the host’s adaptability to new niches through AMGs in resources-limited condition, indirectly increasing the potential of ARGs proliferation [27]. Furthermore, our validation experiments also demonstrated that phage-encoded AMGs enhanced hosts’ capacity for complex carbohydrate metabolism.

Our research redefines the ecological role of phages in soil. Under the selective pressure induced by MPs, phage-host interactions shift from simple predation toward evolutionary mutualism. The proliferation of ARGs under MPs stress could be considered as an evolutionary byproduct of host adaptation to anthropogenic carbon disturbances. In this context, phages employ MPs-induced carbon bioavailability-dependent strategies, mediating ARGs transfer through lysogenic conversion under conditions of higher carbon bioavailability, and encoding AMGs to enhance fitness of ARGs-carrying taxa under low carbon bioavailability conditions. These phages-mediated processes underscore that phages serve as a critical modulator of soil resistome, bridging the gap between anthropogenic carbon perturbation and antibiotic resistance evolution. Understanding these phages-driven adaptation mechanisms is essential for predicting resistance trajectories and developing management frameworks that account for phage community functions in increasingly plastic-impacted terrestrial ecosystems.

## Conclusion

In this study, we proposed a hypothetical framework for phage-host interaction-mediated ARG alterations. Our results revealed the distinct microbial contributions to the soil ARG enrichments upon biodegradable and nondegradable MPs exposure. Biodegradable MPs could trigger SOS response and MGE-mediated horizontal transfer, while lysogenic phages synergistically promote ARG transduction. In contrary, nondegradable MPs might selectively enriched the ARGs-carrying bacteria harboring genes for carbon degradation and phage-encoded AMGs, enhancing their competitive fitness. These findings reveal how bacteria and phages cooperatively reshape microbial functional traits under MPs stress. Biodegradable and nondegradable MPs appeared to be associated with distinct patterns of ARGs enrichment, potentially reflecting divergent underlying processes. Furthermore, by providing insights into mutualistic phage-host evolution under divergent carbon availability, our findings offer an additional perspective on viral strategies potentially contributing to soil resistome expansion, with potential implications for assessing MP-associated ARG risks. Future work should extend these findings across diverse systems, as natural soil ecosystems encompass additional complexities that may modulate phage-host interactions and ARG profiles.

## Materials and methods

### Experimental design and sample collection

To explore the long-term effect of MPs on soil ARGs, the field experiment was conducted in a remote, minimally disturbed grassland in Hulunbuir, China (119.38E, 50.20 N) since 2020. Different MPs were added at an environment-relative concentration of 0.5% (v/v) [40, 54]. The MPs were selected based on environmental relevance [55, 56], including four biodegradable MPs [PLA, PHA, PBS, and PBAT] and four nondegradable MPs [PVC, PS, PP, and POM]). All experimental treatments were performed in triplicate to ensure statistical reliability. After 2-year incubation, soil samples were collected and stored at −80°C for DNA and RNA extraction, while other soil samples were stored at 4°C for physicochemical properties measurement, including total carbon, total nitrogen, dissolved organic carbon, and soil organic carbon.

### DNA extraction, 16S rRNA sequencing, metagenomics, and viromics

The total DNA was extracted from each sample (0.5 g) using the FastDNA Spin Kit (MP Biomedicals, USA) according to the manufacturer’s instructions, with a final volume of 30 μL. DNA quality and concentration were determined by 1.0% agarose gel electrophoresis and a NanoDrop 2000 spectrophotometer (Thermo Scientific, USA). The details of 16S rRNA sequencing were provided in Supplementary Text S1. For metagenomics sequencing, libraries were constructed using Tecan Ovation Ultralow System V2 and then sequenced on the NovaSeq 6000 system (Illumina, San Diego, CA) to obtain 150 bp paired-end reads. After processing with Trimomatic, the clean data was assembled into contigs based on the De Bruijn graph algorithm [57]. Assembled contigs longer than 500 bp were then used to predict open reading frames and translated into amino acid sequences using Prodigal [58]. A non-redundant gene catalog was generated with CD-HIT at a 95% identity and 90% coverage threshold [59], with the longest sequences selected as representatives. The abundance of genes in each sample was calculated using BBMap by mapping the genes to clean data. Vamb package was used for binning with default parameters, with CheckM [60] and GTDB-Tk [61] were used for estimation of MAG quality (≥50% completeness, ≤10% contamination) and annotation, respectively. Gene functions annotation was performed with DIAMOND by comparing to KEGG [62], eggNOG [63] and CAZy [64] databases.

Virus-like particles were purified and concentrated as described by previous studies (Text S2) [26, 65]. Virome DNA was extracted with MiniBEST Viral RNA/DNA Extraction Kit Ver 5.0 (Takara Bio Inc., China). A macrovirus community sequencing library was generated using the NEB Next Ultra DNA Library Prep Kit for Illumina. The sequencing and data process were the same as in metagenomics. Assembly contigs >5 kb were used for further analysis. VirSorter2, Deepvirfinder, and VIBRANT were applied for screening the candidate viral sequences [66–68]. The criteria were based on previous study [69]. The vOTUs were obtained by clustering candidate phage sequences using the MUMmer with ≥85% sequences length and ≥ 95% similarity. The longest sequence was considered as the representative sequence. CheckV was used for assessing the completeness and contamination of vOTUs, and bwa was then applied for vOTU abundance calculation [70]. The phage-host interactions were predicted with three methods: CRISPR (≥95% identity and ≤ 2 single nucleotide polymorphisms), tRNA (≥95% identity), and genomic homology match (bitscore ≥50, e-value ≤10–3, identity ≥70%, matching length ≥ 2500 bp) [71, 72]. Phage hosts obtained by each method were combined and retained to analyze the host range comprehensively. Lysogenic phages were identified by Deephage using 0.6 as the cutoff [73]. DRAMv (auxiliary score: 1–3, amg flags: -M and -F) combined with VIBRANT (default parameters) were used for AMG identification [67, 74].

### ARG identification and metatranscriptomics analysis

To identify ARG encoded by bacteria and phages, clean reads were annotated using the ARGs-OAP 3.2 pipeline with reference to the curated databases: the Structure Antibiotic Resistance Gene database (SARG) and the MobileOG database. Putative bacterial ARGs were identified using thresholds of sequence identity ≥80%, coverage ≥70%, and e-value <1e–5 [75]. Given the substantially higher evolutionary rate and frequent genetic recombination in phage genomes [76, 77], a lower identity threshold (≥ 60%) was applied for phage-encoded ARGs [22]. The risk levels of identified ARGs were classified as Rank I or Rank II according to previously established criteria [46]. The MGE were annotated with MGE marker gene database mobile-OG-db80 [78], applying the same thresholds as those used for ARG annotation.

The metatranscriptome data was used to assess the transcriptional activity of ARGs and functional genes. The relative transcriptional abundance of ARGs was quantified using CoverM pipeline (v0.61). Metatranscriptomic reads were quality-filtered using Trimmomatic(v0.36) [79]. CD-HIT was employed for redundancy removal with default parameters. The remained mRNA reads were mapped backed to ARG sequences to identify gene activity based on the average coverage of transcripts using minimap2 of the CoverM pipeline [21]. Specifically, quality-controlled reads were initially aligned to viral contigs using the “make” command to generate BAM files. The “filter” command was applied to exclude low-quality alignments (read identity ≤95% and aligned percent ≤75%), with the latter threshold relaxed for viral sequences to account for their higher divergence and fragmentation [80]. Quality-controlled metatranscriptomic reads were mapped to ARG sequences using CoverM to calculate “tpm,” “rpkm” and “count.” ARGs were considered transcriptionally active when tpm > 0 in both duplicates of at least two out of three biological replicates. ARGs encoded by phages were deemed as active even if one viral gene exhibited tpm > 0 [21, 81]. The transcriptional activity of functional genes, including oxidative stress and complex carbohydrate degradation, were calculated with salmon [82].

### Laboratory experiments for enhanced ARGs dissemination by phage through different strategies

To validate that lysogenic phage facilitates ARGs transduction under biodegradable MPs treatments, soil bacterial and viral suspension were extracted from biodegradable MPs-treatments soil and CK. In detail, 100 g of soil was mixed with 100 mL of M9 medium and shaken at 250 rpm for 2 h. The mixture was centrifuged at 8000 rpm for 20 min. The supernatant was filtered through a 0.22 μm membrane by tangential flow filtration. Mitomycin C was added to bacterial suspensions with particles >0.22 μm and incubated in the dark for 24 h. The lysogenic phages induced by Mitomycin C were concentrated by tangential flow filtration through a 100 kDa sterile filter (Sartorius, Germany). Half volume of the lysogenic phages was inactivated at 121°C for 1 h [83–85]. For the incubation, 1 mL bacterial suspension and 3 mL phage extract were mixed in 10 mL sterile tubes and incubated in the dark at 25°C for 3 days. This phage-excess setup was used to maximize phage-host encounters and favor phage infection, and preventing excessively high phage inputs from inducing artificial lysogeny that could interfere with ARG transduction detection [86, 87]. Six treatments (3 phage treatments × 2 soil types) replicated 4 times were established as follows: (1) bacterial suspension and M9 medium (CK); (2) bacterial suspension and lysogenic phage extract (BP); (3) bacterial suspension and inactivated lysogenic phage extract (BIP). At the end of incubation, DNA was extracted from 1 mL of sample using TIANamp Genomic DNA Kit (Tiangen Biotech, China) according to the instructions, with a final volume of 100 μL.

Phages can enhance the host adaptation to new niches through AMGs, indirectly intensifying the accumulation and spread of ARGs [27]. Therefore, to verify the influence factors underlying ARGs enrichment under nondegradable MPs exposure, we synthesized a candidate AMG and experimentally tested its ability to assist the host in degrading complex compounds. In our research, S8C1281 exhibited the highest abundance among ARG-carrying bacteria, its associated phage-encoded gene for polysaccharide degradation (PF04616) was selected to investigate whether phage assist host carbon utilization. The target gene was synthesized by PCR-based accurate synthesis. The synthesized fragment was digested with restriction enzymes and ligated into the pET-28a vector and transformed into *E. coli* BL21 (DE3). Positive clones were selected on LB agar containing kanamycin (50 μg/mL) and verified by colony PCR, in which a small number of bacterial cells from each colony was directly added to the PCR reaction mixture as template. The recombinant plasmid pET-28a-PF04616 was transformed into *E. coli* BL21 (DE3) for protein expression, induced with 0.2 mM IPTG at 37°C for 4 h (200 rpm). *E. coli* BL21 (DE3) harboring the empty pET-28a plasmid was used as a control. The cultivation experiments were conducted in media with xylan as the sole carbon source, with the xylan concentrations being 0.5, 1, 2, and 4 mg/mL, respectively. The bacterial solution was mixed with these sterile media in triplicate at a ratio of 1:100 (37°C, 180 rpm and 48 h). The content of xylose was detected with UV–vis spectrophotometer to quantify the degree of xylan degradation. The OD_600_ was measured before and after the experiments.

### Statistical analysis

Relationships among the data were examined through linear fitting with “ggplot2” packages. Random forest analysis was performed to analyze the contributions of MGE, bacterial dissimilarity (based on 16S rRNA), soil properties to ARGs enrichments. Multiple co-inertia analysis (MCOA) was applied to integrate the information from four databases (life history traits, KEGG, CAZy, and eggNOG) to identify the multivariate axes that best explained the aggregation patterns of the soil bacterial metagenomic community under MPs exposure. Normality of data distribution was assessed using the Shapiro–Wilk test in SPSS 26.0. One-way ANOVA with Tukey’s HSD post hoc test and Student’s *t* test were used for parametric data; the Wilcoxon rank-sum test was used for non-parametric data. Data visualizations were generated using Chiplot (https://www.chiplot.online/) and Origin 2023b.

## Supplementary Material

Supplementary_material_wrag258

## Data Availability

The amplicon sequencing, metagenomic sequencing, viromic sequencing, and metatranscriptomic sequencing data produced in this study were deposited in the National Center for Biotechnology Information with accession numbers PRJNA1146707, PRJNA1146756, PRJNA1146757, and PRJNA1146720.
