# Supplementary material for: Microplastic-driven carbon availability differentiates phage-host interactions in shaping soil resistome
Source: ISME J. 2026 Oct 6;20(1):wrag258. doi: 10.1093/ismejo/wrag258 (PMC13704784; doi:10.1093/ismejo/wrag258)
Supplement: Supplementary file 1 [file supplementary_material_wrag258.zip › supporting.docx]

**Microplastic-driven carbon availability differentiates phage-host interactions in shaping soil resistome**

Short title: Contrasting carbon drive resistome

Lihong Xie ^a, #^, Lu Wang ^b, c, #,^ *, Lijuan Ma ^b^, Jie Wang ^b^, Di Wu ^d^, Aijun Lin ^a^, Leyun Wang ^a^, Mao Ye ^e^, Xiujin Li ^a^, Tingting Zhang ^a,^ *, Dong Zhu ^b, c,^ *

a Department of Environmental Science and Engineering, College of Chemical Engineering, Beijing University of Chemical Technology, Beijing 100029, China

b State Key Laboratory of Regional and Urban Ecology, Institute of Urban Environment, Chinese Academy of Sciences, Xiamen 361021, China

c Zhejiang Key Laboratory of Pollution Control for Port-Petrochemical Industry, CAS Haixi Industrial Technology Innovation Center in Beilun, Ningbo 315830, China

d Institute for Global Food Security, School of Biological Sciences, Queen’s University Belfast, 19 Chlorine Gardens, Belfast, BT9 5DL, United Kingdom

e National Engineering Research Center for Soil Nutrient Management and Pollution Remediation, Institute of Soil Science, Chinese Academy of Sciences, Nanjing 210008, China

\# Lihong Xie and Lu Wang contributed equally to this work.

* Corresponding author:

Dong Zhu ([dzhu@iue.ac.cn](mailto:dzhu@iue.ac.cn)), Tingting Zhang ([zhangtt@mail.buct.edu.cn](mailto:zhangtt@mail.buct.edu.cn)), Lu Wang (luwang@iue.ac.cn)

**This file includes:**

**Supplementary Text S1-S2**

**Supplementary Fig. S1-S12**

**Text S1 16S rRNA sequencing**

The V4-V5 regions of bacterial 16S rRNA genes were amplified using primers 515F (GTGCCAGCMGCCGCGG) and 907R (GGACTACNVGGGTWTCTAA) [1]. The PCR reaction mixture including 4 μL 5 × Fast Pfu buffer, 2 μL 2.5 mM dNTPs, 0.8 μL each primer (5 μM), 0.4 μL Fast Pfu polymerase, 10 ng of template DNA, and ddH_2_O to a final volume of 20 µL. PCR amplification cycling conditions were as follows: initial denaturation at 95 ℃ for 3 min, followed by 27 cycles of denaturing at 95 ℃ for 30 s, annealing at 55 ℃ for 30 s and extension at 72 ℃for 45 s, and single extension at 72 ℃ for 10 min, and end at 4 ℃. The PCR product was extracted from 2% agarose gel and purified using the PCR Clean-Up Kit (YuHua, Shanghai, China) according to manufacturer’s instructions and quantified using Qubit 4.0 (Thermo Fisher Scientific, USA). Purified amplicons were pooled in equimolar amounts and paired-end sequenced on an Illumina Nextseq2000 platform (Illumina, San Diego,USA) according to the standard protocols by Majorbio Bio-Pharm Technology Co. Ltd. (Shanghai, China). The analysis of 16S rRNA sequences was carried out by Qiime2 (v2022.0671) [2]. Raw pair-end reads were filtered, and the chimeras were removed using the DADA2 plugin. Amplicon sequence variants (ASVs) were clustered with 100% similarity based on the representative sequences. Bacterial sequences were classified using SILVA Release138 (<http://www.arb-silva.de>).

**Text S2 Purification of virus-like particles**

Soil samples were suspended in 1.0% potassium citrate buffer and homogenized. After low-speed centrifugation (7,000 × g, 10 min, 4 °C) to remove soil debris and bacterial cells, the supernatant was passed through a 0.22 μm hydrophilic PTFE membrane. The filtrate was subsequently concentrated using tangential flow filtration (TFF, Sartorius, Germany) [3]. Phage particles were further precipitated by polyethylene glycol 8000 to a final concentration of 10% (w/v), followed by overnight incubation at 4 °C and centrifugation (8,000 × g, 30 min). The resulting virus-like particles were resuspended in TE buffer and treated with DNase I and RNase A. Purified virus-like particles were enumerated by SYBR Gold staining and epifluorescence microscopy to estimate phage abundance and the virus-to-bacteria ratio.

Fig. S1 The profile of soil ARGs across MPs treatments, colored by ARGs types. The outer and inner circle represent the ARGs type and subtype, respectively. MLS indicated the gene encoding resistance to macrolide-lincosamide-streptogramin.

Fig. S2 The transcriptional activity of ARGs, as well as the number and abundance of active ARGs in soil bacteria (A) and phage (B).

Fig. S3 The total abundance of Rank-I (A) and Rank-II (B) high-risk ARGs in soil bacteria. Lowercase letters above the box and stack bar chart indicate significant differences among different treatment levels in Tukey’s post hoc tests (p < 0.05). p values among CK, biodegradable and nondegradable MPs treatments were calculated using t test. Significance among treatments is denoted as * *p*<0.05, ** *p* < 0.01.

Fig. S4 (A) Relationship between MGE and ARGs. (b)The abundance of MGE under different treatments. Lowercase letters above the box and stack bar chart indicate significant differences among different treatment levels in Tukey’s post hoc tests (*p* < 0.05). *p* values among CK, biodegradable and nondegradable treatments were calculated by the pairwise *t* test. Significance among treatments is denoted as * P<0.05.

Fig. S5 The soil physicochemical properties: (A) DOC, (B) SOC and (C) C/N

Fig. S6 The bacterial composition at phylum level across all treatments

Fig. S7 The relative abundance of bacteria at family level, with significant change with MPs additions is shown on the right, calculated by their average abundance in treatments minus CK.

Fig. S8 Variable contributions to MCOA1 and MCOA2, the information was shared by life history traits and eggNOG datasets. Only the most important variables with significant correlation (*p*<0.05) with MCOA1 and MCOA2 are presented in Fig.

Fig. S9 Response of bacterial community (transcriptional activity of complex compounds degradation gene) to carbon availability.

Fig. S10 The abundance (A) and transcriptional activity (B) of genes related ROS, SOS response and conjugative transfer. *p* values between CK and MPs treatments were calculated by the pairwise *t* test, * *p*<0.05.

Fig. S11 The composition of phage community at family level (A). Alpha diversity of phage community (B). Response of phage community response to carbon availability (C).

Fig. S12 The proportion of ARGs encoded by phage with different life style

Fig. S13 The linear correlation between phage and its host under nondegradable MPs

Fig. S14 Abundance variations of ARGs-encoding MAGs (compared to CK, outer circle), phylum-level distribution of ARG-encoding MAGs with increased abundance (inner circle) under (a) biodegradable and (b) nondegradable MPs treatments.

Fig. S15 The number and transcriptional activity of carbon-related gene annotated in KEGG (A, C) and CAZy (B, D) databases. *p* values between ARG-carrying bacteria and nARG-carrying bacteria were calculated by the Wilcoxon rank-sum test. Significance among treatments is denoted as * *p*<0.05, ** *p*<0.01, *** *p*<0.001.

**Reference:**

1.	Zhou X, Liu X, Liu M *et al.* Comparative evaluation of 16s rrna primer pairs in identifying nitrifying guilds in soils under long-term organic fertilization and water management. *Frontiers in Microbiology*. 2024; **15 - 2024**

2.	Shen L-Q, Wang L, Yao Z *et al.* Phages drive the dissemination of antibiotic resistance genes by facilitating host adaptation to heavy metal stress. *Proceedings of the National Academy of Sciences*. 2026;**123**:e2535653123 <https://doi.org/10.1073/pnas.2535653123>

3.	Zheng X, Jahn MT, Sun M *et al.* Organochlorine contamination enriches virus-encoded metabolism and pesticide degradation associated auxiliary genes in soil microbiomes. *The ISME Journal*. 2022;**16**:1397 <https://doi.org/10.1038/s41396-022-01188-w>
